# Supplementary material for: Hybrid Models and Biological Model Reduction with PyDSTool
Source: PLoS Comput Biol. 2012 Aug 9;8(8):e1002628. doi: 10.1371/journal.pcbi.1002628 (PMC3415397; doi:10.1371/journal.pcbi.1002628)
Supplement: Text S4 — Complete source code for the PyDSTool package (version 0.88.120504). Includes API documentation and help files linking to web pages. This file is identical to the current public release on Sourceforge.net. (ZIP) [file pcbi.1002628.s004.zip › PyDSTool/html/PyDSTool.Toolbox.phaseplane.fixedpoint_nD-class.html]

xml version="1.0" encoding="ascii"?


PyDSTool.Toolbox.phaseplane.fixedpoint\_nD


| Home | Trees | Indices | Help | PyDSTool |
| --- | --- | --- | --- | --- |

Package PyDSTool ::
Package Toolbox ::
Module phaseplane ::
Class fixedpoint\_nD

[hide private]

[frames] | no frames]

# Class fixedpoint\_nD

source code

```
object --+
         |
        fixedpoint_nD
```

Known Subclasses:
:   - fixedpoint\_2D

---


|  |  |
| --- | --- |
| Instance Methods | [hide private] |

|  |  |
| --- | --- |
| \_\_init\_\_(self, gen, pt, coords=None, jac=None, description=`'``'`, normord=2, eps=1e-12)  pt must have same dimension as generator, but if a sub-system is being analyzed then specify the sub-system's variables using the coords argument. | source code |

|  |  |
| --- | --- |
| \_get\_eigen(self) | source code |

|  |  |
| --- | --- |
| \_ensure\_jac(self, jac) | source code |

|  |  |
| --- | --- |
| \_\_getitem\_\_(self, k) | source code |

|  |  |
| --- | --- |
| \_\_setitem\_\_(self, k, v) | source code |

|  |  |
| --- | --- |
| \_classify(self) | source code |

**Inherited from `object`**:
`__delattr__`,
`__getattribute__`,
`__hash__`,
`__new__`,
`__reduce__`,
`__reduce_ex__`,
`__repr__`,
`__setattr__`,
`__str__`


|  |  |
| --- | --- |
| Class Variables | [hide private] |

\_classifcations = `('spiral', 'node', 'saddle')`

\_stability = `('s', 'c', 'u')`


|  |  |
| --- | --- |
| Properties | [hide private] |

**Inherited from `object`**:
`__class__`


|  |  |
| --- | --- |
| Method Details | [hide private] |

|  |  |
| --- | --- |
| \_\_init\_\_(self, gen, pt, coords=None, jac=None, description=`'``'`, normord=2, eps=1e-12)  *(Constructor)* | source code |

pt must have same dimension as generator, but if a sub-system is being
analyzed then specify the sub-system's variables using the coords
argument.

Overrides: object.\_\_init\_\_

  


| Home | Trees | Indices | Help | PyDSTool |
| --- | --- | --- | --- | --- |

|  |  |
| --- | --- |
| Generated by Epydoc 3.0.1 on Fri May 4 15:24:09 2012 | http://epydoc.sourceforge.net |
